# Multiple Sites Ultrasonography of Peripheral Nerves in Differentiating Charcot–Marie–Tooth Type 1A from Chronic Inflammatory Demyelinating Polyradiculoneuropathy

**DOI:** 10.3389/fneur.2017.00181

**Published:** 2017-05-04

**Authors:** Jingwen Niu, Liying Cui, Mingsheng Liu

**Affiliations:** ^1^The Department of Neurology, Peking Union Medical College Hospital, Chinese Academy of Medical Sciences, Beijing, China

**Keywords:** Charcot–Marie–Tooth disease, chronic inflammatory demyelinating polyradiculoneuropathy, nerve ultrasonography, cross-sectional area, diagnosis

## Abstract

**Introduction:**

Multiple sites measurement of cross-sectional areas (CSA) by ultrasound was performed to differentiate Charcot–Marie–Tooth type 1A (CMT1A) and chronic inflammatory demyelinating polyradiculoneuropathy (CIDP).

**Methods:**

Nine patients with CMT1A, 28 patients with CIDP, and 14 healthy controls (HC) were recruited prospectively. Consecutive ultrasonography scanning was performed from wrist to axilla on median and ulnar nerves. CSAs were measured at 10 predetermined sites of each nerve.

**Results:**

CMT1A had significantly larger CSAs at all sites of median and ulnar nerves (*p* < 0.01). In CMT1A, CSAs increased gradually and homogeneously from distal to proximal along the nerve, except potential entrapment sites. CIDP displayed three different morphological patterns, including mild enlargement in 15 patients, prominent segmental enlargement in 12, and slight enlargement in 1, among which different treatment responses were observed. All patients with mild nerve enlargement treated with intravenous immunoglobulin were responsive (7/7), while less than half of those with prominent segmental enlargement (3/7) were responsive (*p* < 0.01).

**Discussion:**

Consecutive scan along the nerve and multiple sites measurement by ultrasound could supply more detailed morphological feature of the nerve and help to differentiate CMT1A from CIDP.

## Introduction

Both Charcot–Marie–Tooth type 1A (CMT1A) and chronic inflammatory demyelinating polyradiculoneuropathy (CIDP) are chronic demyelinating polyneuropathy involving motor and sensory nerve. Early onset, family history, pes cavus, diffuse, and homogeneous nerve-conduction velocity slowing without conduction block suggest diagnosis of CMT1A (1); while recurrent weakness, conduction block, and clinical improvement following immunomodulatory treatment favor CIDP (2). However, differential diagnosis may be hampered by inconspicuous family history, too low or absent compound motor action potential (CMAP) amplitude, or poor response to steroid therapy. The usefulness of peripheral nerve ultrasound in distinguishing CMT1 from CIDP has been reported recently (3–5). The amount of enlargement was more prominent in CMT1 compared to CIDP, as evaluated by ultrasound pattern sum score (UPSS) (5), nerve size index (NSI) (4), or enlargement site number (ESN) (3). Homogeneous enlargement was significantly more often seen in CMT1, while in CIDP the enlargement was regional, homogeneous, or inhomogeneous (5). The description of overall enlargement at anatomical landmarks, the distribution of enlargement, and the pattern differences might help to differentiate acquired and inherited neuropathies (5). However, these studies measured only two to five predetermined sites in one nerve, providing limited morphologic information for the whole nerve. By scanning the whole nerve and measuring cross-sectional areas (CSAs) in 10 consecutive sites in median and ulnar nerves, we got more accurate and thorough morphological information of the nerve.

## Materials and Methods

### Subjects

Between January 2015 and July 2016, 9 patients with CMT1A, 28 patients with CIDP, and 14 healthy controls (HC) were recruited prospectively in Peking Union Medical College hospital. All CMT1A patients had demyelinating features consistent with an inherited polyneuropathy and were ascertained by genetic testing. For the diagnosis of definite CIDP, we used the diagnostic criteria proposed by the Joint Task Force of the European Federation of Neurological Societies and the Peripheral Nerve Society (2). All patients and HC received a clinical neurological examination, nerve ultrasound, and nerve-conduction studies. The ethic committee of Peking Union Medical College Hospital approved our study protocol, and all patients and HCs signed informed consent in accordance with the Declaration of Helsinki.

The mean ages of CMT1A patients, CIDP patients, and HC were 39 years (range 10–57 years), 39 years (range 13–61 years), and 39 years (range 19–52 years), respectively. There were no statistical differences among the age of the three groups. There were 3 males in the CMT1A group, 22 males in the CIDP group, and 8 males in the HC group.

### Ultrasonographic Studies

Ultrasonographic tests were performed *via* nerve tracing from wrist to axilla on median and ulnar nerve with an 8–12 MHz linear array transducer (LOGIQ e, GE company, USA). The initial settings were kept constant during all examinations excluding depth. Ultrasonographic tests were performed bilaterally in most subjects, and only on right side in 3 CMT1A patients, 3 CIDP patients, and 10 HC. The transducer was adjusted to be perpendicular to the nerve to obtain the minimal cross-sectional image. The CSAs at the predetermined sites of each nerve were measured by tracing just inside the hyperechoic rim of the nerve. Ten predetermined sites were measured on each nerve. For median nerve, the 10 sites included the outlet of carpel tunnel, the middle point of wrist crease, the inlet of carpel tunnel, 4 cm proximal to wrist crease, middle between wrist crease and elbow, entrance before into pronator teres, elbow, 4 cm above elbow, 8 cm above elbow, axilla. For ulnar nerve, the 10 sites included wrist, 4 cm proximal to wrist, departing point from ulnar artery, alongside the muscle belly of flexor carpi ulnaris, outlet of flexor carpi ulnaris, outlet of cubital tunnel, inside cubital tunnel, inlet of cubital tunnel, 4 cm proximal to inlet of cubital tunnel, 8 cm proximal to inlet of cubital tunnel. These 10 sites were marked as A1–A10 from distal to proximal separately for median and ulnar nerves. After CSA measurement, the nerves were again traced continuously and recorded thoroughly.

Chronic inflammatory demyelinating polyradiculoneuropathy patients were divided into three subgroups. For CIDP type 1 group, CSAs at all sites of any nerve studied were less than 150% of CSAs at the corresponding sites in HC; for CIDP type 2 group, CSAs at one or more sites of any nerve studied were more than 150% but less than 250% of CSAs at the corresponding sites in HC; for CIDP type 3 group, CSAs at one or more sites of any nerve studied were more than 250% of CSAs at the corresponding sites in HC.

### Statistical Analysis

For statistical analysis, we used IBM SPSS Statistics, version 20. The CSAs of CMT1A and HC showed a normal distribution, and the CSAs of CIDP showed a non-normal distribution (as evaluated by single sample K–S test). Thus, *t*-test was used for evaluating differences of CSA between CMT1A and HC, while Mann–Whitney *U* test was used for comparison between CIDP and the other group. *t*-Test and Fisher’s exact test were used to compare the clinical features between CIDP type 2 and type 3. Receiver operating characteristic (ROC) curve analysis was performed to evaluate the use of CSA measurements to differentiate CMT1A from CIDP. Two-sided *p* values were calculated for all analyses; *p* < 0.05 was considered significant.

## Results

### Comparison of CSA between CMT1A, CIDP, and HC

The CSAs at 10 different sites of median and ulnar nerve in each group were shown in Table 1. The CSA values in CMT1A were significantly higher than in CIDP at all sites of median and ulnar nerve (all *p* < 0.01). The CSA values in CIDP were significantly higher than in HC at all sites of median and ulnar nerve (all *p* < 0.05).

**Table 1:** **CSA (mean ± SD) at different sites of median and ulnar nerves in CIDP, CMT1A, and HC (mm^2^), and the comparison between one another**.

		A1	A2	A3	A4	A5	A6	A7	A8	A9	A10
**Median nerve**											
HC		6.3 ± 1.8	6.3 ± 1.1	5.0 ± 1.0	6.0 ± 1.8	5.7 ± 1.5	5.4 ± 1.4	5.9 ± 1.1	6.5 ± 1.3	6.5 ± 1.4	6.6 ± 1.7
CIDP		7.9 ± 2.1	7.7 ± 1.6	7.9 ± 2.3	8.5 ± 4.2	9.8 ± 10.9	9.4 ± 7.6	11.7 ± 5.3	12.2 ± 6.2	13.4 ± 11.3	11.4 ± 4.6
CMT1A		11.1 ± 4.6	10.7 ± 2.3	13.1 ± 4.8	15.5 ± 4.7	18.9 ± 4.7	18.5 ± 4.8	26.3 ± 8.7	29.1 ± 11.5	26.1 ± 9.5	19.2 ± 7.4
HC vs. CMT1A	*t*	3.858	4.760	6.391	6.411	11.247	9.524	8.933	6.626	7.323	6.034
	*p*	0.001	0.000	0.000	0.000	0.000	0.000	0.000	0.000	0.000	0.000
HC vs. CIDP	*z*	2.680	2.230	5.416	2.761	3.164	3.653	5.059	4.341	4.261	3.559
	*p*	0.007	0.026	0.000	0.006	0.002	0.000	0.000	0.000	0.000	0.000
CIDP vs. CMT1A	*z*	3.326	4.215	4.566	4.690	5.114	4.769	5.336	5.021	4.718	3.482
	*p*	0.001	0.000	0.000	0.000	0.000	0.000	0.000	0.000	0.000	0.000
**Ulnar nerve**											
HC		3.1 ± 1.1	3.3 ± 0.8	4.4 ± 1.0	4.8 ± 1.0	5.1 ± 0.7	5.1 ± 1.4	6.2 ± 1.9	4.7 ± 1.2	4.4 ± 1.3	4.0 ± 0.9
CIDP		4.5 ± 1.3	5.5 ± 2.3	7.4 ± 5.4	8.2 ± 8.2	8.8 ± 7.2	6.8 ± 2.7	7.4 ± 2.2	7.0 ± 3.0	10.8 ± 17.1	9.8 ± 13.8
CMT		7.5 ± 1.7	9.0 ± 2.0	13.9 ± 2.5	15.4 ± 3.7	14.6 ± 3.7	11.8 ± 2.6	11.6 ± 3.7	16.3 ± 4.7	19.6 ± 2.9	19.5 ± 3.1
HC vs. CMT1A	*t*	8.487	10.787	14.009	10.602	7.794	8.786	5.303	9.201	19.040	17.381
	*p*	0.000	0.000	0.000	0.000	0.001	0.000	0.003	0.000	0.000	0.000
HC vs. CIDP	*z*	3.950	4.795	3.966	3.634	2.819	2.602	2.122	3.949	3.833	3.836
	*p*	0.000	0.000	0.000	0.000	0.005	0.009	0.034	0.000	0.000	0.000
CIDP vs. CMT1A	*z*	5.056	5.048	5.100	5.282	3.783	5.101	4.127	5.485	4.830	4.692
	*p*	0.000	0.000	0.000	0.000	0.000	0.000	0.000	0.000	0.000	0.000

*CSA, cross-sectional area; CIDP, chronic inflammatory demyelinating polyradiculoneuropathy; CMT, Charcot–Marie–Tooth disease; HC, healthy control*.

*For median nerve: A1—the outlet of carpel cannel, A2—the middle point of wrist crease, A3—the inlet of carpel cannel, A4—4 cm proximal to wrist crease, A5—middle between wrist crease and elbow, A6—entrance into pronator teres, A7—elbow, A8—4 cm above elbow, A9—8 cm above elbow, A10—axilla. For ulnar nerve: A1—wrist, A2—4 cm above wrist, A3—departing point with ulnar artery, A4—alongside the muscle belly of flexor carpi ulnaris, A5—outlet of flexor carpi ulnaris, A6—outlet of cubital tunnel, A7—inside cubital tunnel, A8—inlet of cubital tunnel, A9—4 cm proximal to inlet of cubital tunnel, A10—8 cm proximal to inlet of cubital tunnel*.

### The ROC of CSAs for Differentiating between CIDP and CMT1A

**Figure 1:**
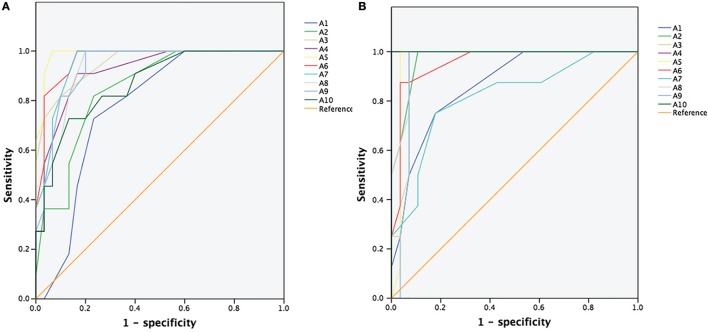
**Receiver operating characteristic (ROC) curve for differentiating chronic inflammatory demyelinating polyradiculoneuropathy from Charcot–Marie–Tooth type 1A**. **(A)** ROC curve of the cross-sectional areas (CSAs) of 10 different sites (A1–A10) of the median nerve. **(B)** ROC curve of the CSAs of 10 different sites (A1–A10) of the ulnar nerve.

Figure 1 showed the ROC curve analyses of the CSAs at different sites of the median and ulnar nerve. Area under curve (AUC) and cutoff values at each site were shown in Table 2. AUCs were above 0.8 for all sites except site A1 in median nerve, and were above 0.9 except sites A5 and A7 in ulnar nerve.

**Table 2:** **The AUC, suggested cutoff values, sensitivity, and specificity of CSA in different sites of median and ulnar nerves in differentiating between CIDP and CMT1A**.

	A1	A2	A3	A4	A5	A6	A7	A8	A9	A10
**Median**										
AUC	0.782	0.885	0.886	0.896	0.935	0.928	0.953	0.939	0.913	0.845
Cutoff values	8.5	8.5	9.5	10.5	12	11.5	17	16.5	14.5	14.5
Sensitivity	0.8	0.846	0.8	0.933	1	1	1	1	1	0.727
Specificity	0.7	0.78	0.882	0.792	0.88	0.827	0.83	0.827	0.745	0.821
**Ulnar**										
AUC	0.922	0.924	0.933	0.946	0.895	0.933	0.849	0.963	0.923	0.922
Cutoff values	5.5	6.5	9.5	9.5	9	8.5	9.5	10.5	11.5	14.5
Sensitivity	0.867	1	1	1	1	0.933	0.8	0.933	1	1
Specificity	0.827	0.788	0.86	0.923	0.848	0.857	0.846	0.923	0.84	0.9

*CSA, cross-sectional area; CIDP, chronic inflammatory demyelinating polyradiculoneuropathy; CMT, Charcot–Marie–Tooth disease; AUC, area under curve*.

### The Pattern of Enlargement in CMT1A and CIDP Patients

In CMT1A patients, gradual and homogeneous enlargement from distal to proximal was observed, with the exception of potential entrapment sites (cubital tunnel for ulnar nerve, as well as carpel tunnel for median nerve).

**Figure 2:**
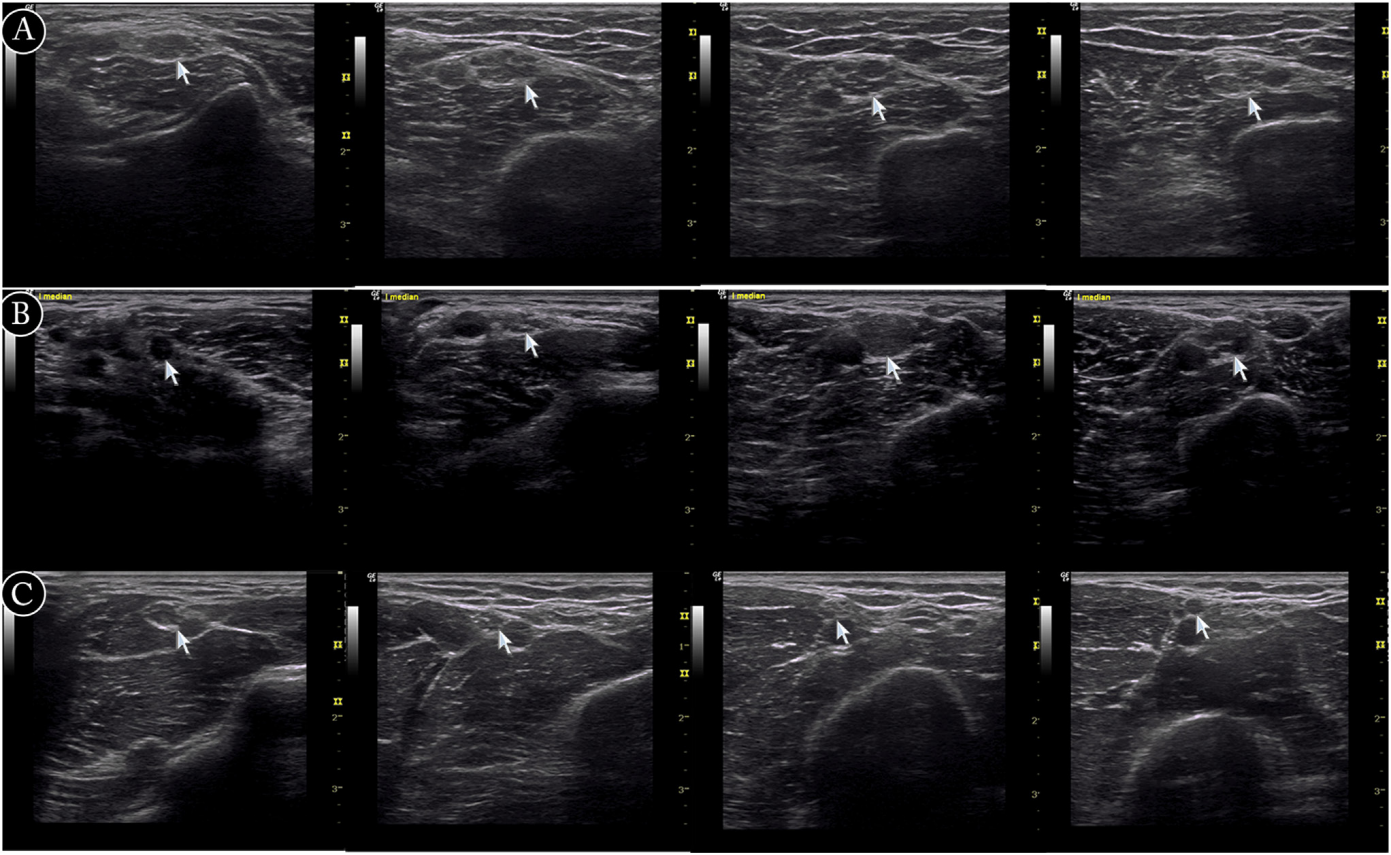
**Examples of ultrasound cross-sections showing measurements of cross-sectional area of median nerve in arm**. **(A)** Homogeneous enlargement was shown at sites A7–A10 of median nerve in a Charcot–Marie–Tooth type 1A patient. **(B)** Segmental enlargement was seen at sites A7–A10 of median nerve in a chronic inflammatory demyelinating polyradiculoneuropathy (CIDP) patient (type 3). **(C)** Mild nerve enlargement was shown at sites A7–A10 of median nerve in a CIDP patient (type 2).

**Figure 3:**
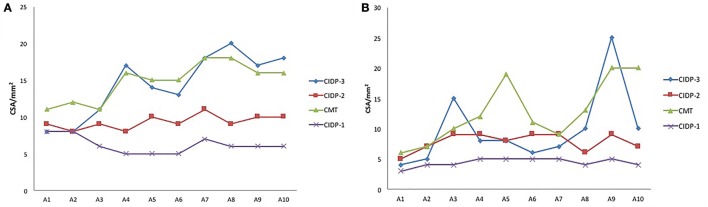
**The cross-sectional area (CSA) at different sites of median (A) and ulnar (B) nerves of four patients**. Three chronic inflammatory demyelinating polyradiculoneuropathy (CIDP) patients of different types, as well as a Charcot–Marie–Tooth type 1A (CMT1A) patient were compared. Segmental prominent enlargement was observed in patient of CIDP type 3, and mild nerve enlargement was observed in CIDP type 2. In CMT1A patient, the CSAs at outlet of cubital tunnel, cubital tunnel, and inlet of cubital tunnel were smaller than adjacent sites.

There were three different types of nerve morphology observed by ultrasound in CIDP patients. Mild nerve enlargement was observed in 15 patients (CIDP type 2), segmentally prominent enlargement in 12 (CIDP type 3, Figure 2), and little enlargement in one patient (CIDP type 1). The comparison of these three types, as well as CMT1A patients, was shown in Figure 3. In CIDP type 3, the CSA may be dramatically enlarged at some sites, and normal at adjacent sites.

We compared the clinical features of CIDP type 2 and CIDP type 3 (Table 3). The two groups were of similar age and gender ratio. Disease duration at the time of examination was longer in CIDP type 3. The percentages of patients of relapsing or progressive pattern were similar in the two groups. The percentages of patients responsive to steroid were similar in the two groups. All patients of type 2 treated with intravenous immunoglobulin (IVIG) were responsive (7/7), while less than half (3/7) of type 3 treated with IVIG were responsive (*p* = 0.002).

**Table 3:** **The comparison of clinical data between chronic inflammatory demyelinating polyradiculoneuropathy (CIDP) type 2 and CIDP type 3**.

	CIDP2	CIDP3	*p*	*t*
Age	41.1 ± 15.7	37.8 ± 13.0	0.422	0.809
Male/female	12/3	10/2	1.000	
Duration (months)	20.7 ± 29.9	36.2 ± 20.8	0.037	0.037
Aggravating/remission period	10/5	5/7	0.099	
Relapsing/progressive	6/8	6/6	0.781	
Steroid effective/ineffective	6/1	9/3	0.684	
IVIG effective/ineffective	7/0	3/4	0.002	
MRC sum	15.3 ± 2.9	15.5 ± 2.5	0.876	0.158

*IVIG, intravenous immunoglobulin; MRC, the British Medical Research Council; MRC sum, the summation of MRC of bilateral abductor digiti minus and abductor pollicis brevis. The Qualitative data were expressed as mean ± SD*.

### Consecutive Scan of the Thorough Nerve

Consecutive observation of the thorough nerve offers more detailed morphological features of the nerve, including the distribution of enlarged sites. The recordings of ulnar nerve in a CMT1A patient, two CIDP patients, and a healthy control were shown in Videos S1–S7 in Supplementary Material.

## Discussion

CMT1 and CIDP are both chronic demyelinating peripheral neuropathy involving motor and sensory nerves. Although many different genetic forms of CMT1 are discovered, it is indicated that molecular diagnosis could be established only in 50–70% CMT patients (1, 6). Lack of family history adds difficulty in making the diagnosis and differential diagnosis. Electrophysiological examinations help the differentiation. CMT1 shows diffuse and homogeneous nerve-conduction velocity slowing, usually without conduction block (1); while CIDP shows inhomogeneous nerve-conduction velocity slowing, and often partial motor conduction block or abnormal temporal dispersion (2). However, in CIDP patients with a long disease duration, the conduction velocity might be universally decreased, and partial motor conduction block is not seen in all patients. Also, in cases when motor CMAP is dramatically decreased (i.e., less than 1 mV), nerve-conduction study is often useless in distinguishing between CIDP and CMT1. Recently, as an emerging new method, peripheral nerve ultrasound is becoming more and more important in the differential diagnosis of peripheral neuropathy. Peripheral nerve ultrasound has been studied to help differentiate CMT1 from CIDP (3–5). It was reported that in CMT1, the nerve enlargement is mostly homogeneous and prominent, while CIDP patients show heterogeneous morphologies, ranging from regional/segmental to inhomogeneous and homogeneous nerve enlargement (3–5). However, most existed studies measured no more than three common sites of one nerve, lacking consecutive observation along the thorough nerve trunk. Since in some CIDP patients, the nerve might enlarge or narrow abruptly, changes such as segmental enlargement might be missed if only three or less sites were detected. Consecutive scanning and multiple-sites-recording along the nerve help to evaluate the nerve more thoroughly, compared with previous methods.

In our study, CSAs increased significantly in CMT1A and CIDP. Generally, The CSA values in CMT1A were significantly higher than those in CIDP. According to the ROC analysis (Table 2), CSAs measured by high resolution ultrasound were useful in distinguishing between CMT1A and CIDP, with high sensitivity and specificity. Furthermore, the patterns of morphological change in nerve ultrasound were different between CMT1A and CIDP. In CMT1A patients, CSAs increased gradually from distal to proximal, and there was no abrupt enlargement except at cubital tunnel of ulnar nerve. In CMT1A patients, duplication in PMP22 gene causes impairment of myelin structure and function in peripheral nerves. Schwann cells and abundant connective tissue around thinly myelinated axons (“onion bulbs”) are the main features of pathology in CMT1A (7). These pathological changes happened uniformly in all nerves during the course of the disease, which was consistent with the uniform changes in nerve ultrasound. According to our results, the CSAs at outlet of cubital tunnel, cubital tunnel, and inlet of cubital tunnel were smaller than adjacent sites in CMT1A patients. This phenomenon has not been reported in previous studies. We presume that this might be due to the narrow anatomic space in cubital tunnel which might cause local ulnar nerve stretched or entrapped during daily life, thus limiting the enlargement of nerve trunk. This might also be true for median nerve at carpal tunnel, but was overlooked due to its location at distal part of the nerve.

Most CIDP patients also had nerve enlargement. Moderate enlargement was observed in the majority of our CIDP patients, while segmental prominent enlargement or no enlargement was shown in others. CIDP is an autoimmune-mediated peripheral nerve disease, with segmental demyelination and remyelination resulting in onion bulb formation and varying degrees of interstitial edema and endoneurial inflammation (7). The clinical presentation of CIDP is variable, including typical (proximal and distal weakness with large-fiber sensory loss), atypical (distal large-fiber sensorimotor neuropathy), and multifocal sensorimotor neuropathy (Lewis–Sumner syndrome) (8, 9). The heterogeneous morphology by ultrasound may reflect both the clinical and pathological heterogeneities of the disease. Clinical features of different CIDP types were compared in this study. The only one patient of type 1 was a 13-year-old female with a disease duration of 5.5 months and was responsive to steroid and IVIG treatment. The mean disease duration of type 3 patients was longer than that of type 2 patients. No patient of type 3 had a disease duration less than 10 months. We assume that long disease duration might be necessary for the repeated demyelination and remyelination to form large CSA. All patients of type 2 treated with IVIG were responsive, while less than half (3/7) of type 3 treated with IVIG were responsive. The differences in clinical features and treatment responses might be due to different pathogenesis in CIDP. Ultrasonography might be a possible marker to guide treatment.

Recently, research on autoantibodies in CIDP has received a boost. Antibodies of the IgG4 isotype targeting the paranodal proteins contactin-1 (CNTN1) and neurofascin-155 (NF155) define specific CIDP subtypes, with characteristic clinical features and poor response to IV immunoglobulin (10, 11). In the research by Ogata et al., marked symmetric hypertrophy of cervical and lumbosacral roots/plexuses was present in anti-NF155 antibody-positive CIDP patients examined by MRI (12). In our study, CIDP type 3 also had marked hypertrophy of peripheral nerves and cervical plexuses, as well as poor response to IVIG. Detection of anti-NF155 and anti-CNTN1 antibodies in different types of CIDP patients might be meaningful and might reveal links among serology, ultrasonography, pathogenesis, and treatment responses.

This study suffers from several limitations. First, the reference values of CSAs of nerve ultrasound have not been established in China, so the data of 14 HC were used as control. We also found that the normal values of HC in China were significantly lower than those published in USA and European. More data in HC should be collected for clinical practice in China. Second, the number of cases was limited. The only one patient of type 1 was a 13-year-old female with a disease duration of 5.5 months. It was difficult to distinguish whether the small CSAs in this patient were due to young age and short duration. Further studies are needed to include more cases. Third, there were also some difficulties in analyzing and presenting the data in CIDP patients, due to the large amount of data and different distribution patterns of enlarged CSA, although it was easy for doctors to differentiate CMT1A from CIDP during ultrasound scan along the nerve, as shown in Figure 2 and Supplemental Material. Analyzing merely the mean value of CSA would definitely overlook some features of CSA change in CIDP. For example, the CSA at the same site of ulnar nerve may significantly increase in one patient but be normal in another. Since CSAs in CMT1A increased gradually from distal part to proximal part, the absolute difference of CSAs between two sites of the same nerve was also large. So when we tried to use intra-nerve variation of the same nerve to differentiate CMT1A from CIDP, the AUC of ROC analysis was low. Although the subjective dividing of CIDP into three types did give us some impression of the distribution patterns of enlargement of CSA during dynamic scanning, the absolute cutoff values for defining the three patterns might change in different studies.

In conclusion, the pattern of CSA enlargement is different in CMT1A and CIDP patients. In CMT1A patients, the CSAs increased gradually from distal to proximal along the nerve, except at potential entrapment sites, as revealed by nerve ultrasound. CIDP patients displayed different patterns, including moderate CSA enlargement, segmental prominent enlargement, and little enlargement. Consecutive scanning along the nerve and multiple sites measurement by ultrasound could supply more detailed morphological information and help to differentiate CMT1A from CIDP, and might guide the treatment of CIDP.

## Ethics Statement

This study was carried out in accordance with the recommendations of the ethic committee of Peking Union Medical College Hospital with written informed consent from all subjects. All subjects gave written informed consent in accordance with the Declaration of Helsinki. The protocol was approved by the ethic committee of Peking Union Medical College Hospital.

## Author Contributions

JN: ultrasonography, acquisition of data, completion of statistical analysis, and drafting of the initial manuscript. LC: study concept and design, and critical revision of the manuscript for important intellectual content. ML: study concept and design, ultrasonography, data review, interpretation of results, revision of the initial draft, and writing of the final manuscript.

## Conflict of Interest Statement

The authors declare that the research was conducted in the absence of any commercial or financial relationships that could be construed as a potential conflict of interest.

## Supplementary Material

Videos S1–S7 **The consecutive scan of ulnar nerve in a healthy control, one Charcot–Marie–Tooth type 1A (CMT1A) patient and 2 chronic inflammatory demyelinating polyradiculoneuropathy (CIDP) patients (CIDP type 2 and 3, respectively)**. Videos 1 and 2, the scan of right ulnar nerve in a healthy control (video 1—the segment from wrist to above elbow; video 2—the segment from above elbow to axilla). Video 3, in the CMT1A patient, cross-sectional areas increased gradually from distal to proximal along the nerve, but decreased in the cubital tunnel. Videos 4 and 5, in a patient of CIDP type 3, prominent segmental enlargement of ulnar nerve was observed, especially in the arm (video 4—the segment from wrist to elbow; video 5—the segment from elbow to axilla). Videos 6 and 7, mild nerve enlargement in a patient of CIDP type 2 (video 6—the segment from wrist to above elbow; video 7—the segment from above elbow to axilla).

## Abbreviations

CSA, cross-sectional area; CMT1A, Charcot–Marie–Tooth type 1A; CIDP, chronic inflammatory demyelinating polyradiculoneuropathy; HC, healthy control; IVIG, intravenous immunoglobulin; CMAP, compound motor action potential; UPSS, ultrasound pattern sum score; NSI, nerve size index; ESN, enlargement site number; ROC, receiver operating characteristic; AUC, area under curve; CNTN1, contactin-1; NF155, neurofascin-155.
